# Adolescents’ Knowledge and Attitudes Toward Attention-Deficit/Hyperactivity Disorder in Greek Secondary Schools

**DOI:** 10.3390/pediatric18010026

**Published:** 2026-02-05

**Authors:** Angeliki Giannakea, Vicky Nanousi, Voula Chris Georgopoulos

**Affiliations:** 1 Department of Speech & Language Therapy, University of Patras, 26504 Rion, Greece; 2 Primary Healthcare Laboratory, School of Health Rehabilitation Sciences, University of Patras, 26504 Rion, Greece

**Keywords:** attention-deficit/hyperactivity disorder (ADHD), adolescents, secondary school students, attitudes, educational support

## Abstract

Background/Objectives: Adolescence is a critical developmental period during which peer attitudes and school experiences play an important role in social inclusion and academic adjustment. Although attention-deficit/hyperactivity disorder (ADHD) is common in secondary school populations, adolescents’ own knowledge and attitudes toward ADHD remain underexplored, particularly in non-Anglophone contexts. This study aimed to assess knowledge and attitudes toward ADHD among Greek secondary school students, focusing on awareness of the disorder, perceptions of ADHD-related classroom behaviors, and views on educational support and intervention. Methods: A cross-sectional survey was conducted among 154 adolescents aged 12–18 years attending Gymnasium (Grades 7–9) and Lyceum (Grades 10–12) in Greece. Data were collected using an anonymous online questionnaire assessing prior awareness of ADHD, perceptions of classroom behaviors associated with ADHD, attitudes toward inclusion and teacher support, and views on educational and therapeutic interventions. Adolescents with and without a self-reported ADHD diagnosis completed different questionnaire sections according to study design. Descriptive statistics and chi-square tests were used for data analysis. Results: Approximately two thirds of participants (66.9%) reported prior awareness of ADHD. Among typically developing adolescents (*n* = 134), 83.0% recognized distractibility due to external noise, 70.4% noted off-topic interruptions, and 60.0% reported peers getting up without permission. While 75.5% believed students with ADHD can participate in the classroom, 65.9% also reported academic challenges such as incomplete homework or lower performance. Overall, 79.2% of participants stated that school success depends on teacher and specialist support; however, among adolescents with ADHD (*n* = 20), only 60.0% endorsed this, with 40.0% emphasizing personal effort. Speech-language therapy was viewed as helpful by 55.6% of typically developing adolescents, though 76.9% of adolescents with ADHD reported not receiving such services. Conclusions: Greek adolescents demonstrate moderate awareness of ADHD and generally supportive attitudes toward peers with ADHD, alongside some uncertainty regarding available educational supports. Schools may represent an important context for improving adolescents’ mental health literacy and understanding of ADHD-related support options.

## 1. Introduction

Attention-deficit/hyperactivity disorder (ADHD) is a neurodevelopmental disorder characterized by a persistent pattern of inattention and/or hyperactivity-impulsivity that interferes with functioning or development [1]. Recent umbrella-level evidence indicates that ADHD affects approximately 8% of children and adolescents worldwide [2], with age-stratified meta-analytic estimates of 7–8% in children and 5–6% in adolescents [3]. It is associated with academic underachievement, behavioral difficulties, and challenges in social functioning, which often become more pronounced during secondary school years [4]. Symptoms frequently persist into adolescence, a developmental period marked by increasing academic demands, greater expectations for self-regulation, and heightened sensitivity to peer evaluation. Consequently, adolescents with ADHD often experience difficulties that extend beyond core symptoms, affecting academic performance, social relationships, and emotional well-being.

Adolescence is a critical stage for social development, during which peer acceptance, friendship formation, and social identity play central roles. Adolescents with ADHD are at increased risk for peer rejection, bullying, and social exclusion compared with their peers without ADHD [5]. These social difficulties may arise from behaviors such as impulsivity, inattentiveness, and emotional dysregulation, which are frequently misinterpreted by peers as intentional or disruptive rather than as manifestations of a neurodevelopmental condition. Social challenges during adolescence are particularly consequential, as they have been associated with poorer mental health outcomes, reduced school engagement, and diminished quality of life. Adolescents with ADHD also experience persistent difficulties in friendship formation and maintenance, which have been linked to emotional dysregulation and peer misunderstanding [6].

The social experiences of adolescents with ADHD are strongly influenced by stigma, which encompasses stereotypes, prejudicial attitudes, and discriminatory behaviors operating at individual, interpersonal, and structural levels [7]. ADHD-related stigma has been shown to negatively affect social inclusion, help-seeking behaviors, treatment adherence, and self-esteem across the lifespan [8,9]. Adolescents with ADHD may internalize these negative societal beliefs, which have been linked to increased psychological distress and poorer functional outcomes. Their views are shaped by the attitudes of parents and peers, with evidence showing that parental stigma can contribute to adolescents’ own negative perceptions of ADHD [10]. Peer dynamics also play a critical role during this period, shaping social comparison, acceptance, and identity.

Knowledge and understanding of ADHD are central to shaping these perceptions. While increased visibility has raised awareness, misconceptions remain common. Improved mental health literacy is linked to more inclusive attitudes [8,9], yet recent cross-cultural studies show persistent gaps. For instance, a large study in Lebanon revealed widespread misunderstandings about ADHD, highlighting the need to consider cultural and regional factors when examining adolescent perspectives [11]. Social media platforms, where ADHD is often portrayed in oversimplified or misleading ways, may further blur diagnostic boundaries and encourage self-diagnosis among youth [12]. These dynamics highlight the importance of directly assessing adolescents’ knowledge and attitudes in diverse settings.

Despite the relevance of peer perceptions during adolescence, much of the existing literature on ADHD knowledge and related beliefs has focused on adults, including parents, teachers, and healthcare professionals. Adolescents themselves remain underrepresented in this research, even though peer attitudes are critical determinants of social inclusion and school adjustment. Emerging evidence suggests that adolescents’ beliefs about ADHD are heterogeneous and may differ substantially from adult perspectives, with implications for stigma, coping strategies, and peer relationships [13,14]. Adolescents may rely more heavily on peers, personal experiences, and social media rather than clinical or parental frameworks when forming these beliefs, reflecting the central role of peer networks and digitally mediated information in adolescent health-related knowledge acquisition [15]. For example, adolescents with ADHD have been found to view the condition as less enduring, less biologically based, and more psychosocially influenced than their parents, with such beliefs linked to differences in coping and well-being [13]. At the same time, qualitative research indicates substantial variability in adolescents’ personal perceptions of ADHD: while some emphasize frustration, academic challenges, and social difficulties, others identify perceived strengths and positive traits, reflecting a non-uniform set of experiences and interpretations [14].

The school environment represents a central context in which ADHD-related challenges, perceptions, and interventions converge. Teachers’ knowledge of ADHD and their attitudes toward inclusion have been shown to influence both academic and social outcomes for students with ADHD [16,17]. In addition, evidence from systematic reviews and meta-analyses demonstrates that school-based interventions can improve academic performance, social skills, and overall functioning among children and adolescents with ADHD [4].

Although ADHD is not classified as a primary speech or language disorder, evidence indicates that some children and adolescents with ADHD experience pragmatic and discourse-level communication difficulties, including challenges in narrative skills and social communication. Difficulties in attention and executive functioning can affect listening comprehension and expressive language use within educational settings. As a result, speech-language therapy may form one component of school-based, multidisciplinary support, particularly when communication difficulties contribute to classroom or social challenges [18].

In Greece, existing research has primarily focused on educators’ knowledge and attitudes toward ADHD, revealing gaps in training and preparedness within school settings [16,19]. However, empirical data on adolescents’ own knowledge of ADHD, their perceptions of ADHD-related behaviors, and their views regarding educational support and intervention remain limited. Given cultural, educational, and systemic differences across countries, context-specific evidence is needed to inform targeted awareness initiatives and school-based strategies. Adolescence represents a critical developmental period for the formation of attitudes, social identities, and interpersonal relationships, making it particularly relevant for the examination of ADHD-related perceptions [20].

The present study aimed to assess knowledge and attitudes toward ADHD among Greek adolescents using a cross-sectional survey design. Specifically, it examined adolescents’ awareness of ADHD, their perceptions of behaviors associated with the disorder, and their views regarding educational support and intervention. The study also considered perspectives from both adolescents with and without a reported ADHD diagnosis. By providing detailed baseline data on ADHD-related knowledge and perceptions in this population, the study seeks to inform future educational and awareness initiatives within school contexts.

## 2. Materials and Methods

This study employed a cross-sectional survey design to assess adolescents’ knowledge and attitudes toward ADHD at a single point in time. A cross-sectional approach was considered appropriate given the descriptive nature of the research aims, which focused on awareness, perceptions, and views regarding educational support rather than causal relationships.

The sample consisted of 154 adolescents aged 12–18 years residing in Greece. Participants were recruited using convenience and snowball sampling. Parents were initially contacted by the research team through email and social media platforms. Those who provided informed consent were invited to share the anonymous questionnaire link with their adolescent children and were also encouraged to forward it within their personal networks. Adolescents completed the questionnaire online, independently and anonymously, after providing their own assent. Data were collected between 2 May 2023 and 27 May 2023, primarily in the wider region of Patras.

Inclusion criteria applied to adolescent participants and were as follows:

(a) Age between 12 and 18 years;
(b) Sufficient knowledge of the Greek language to complete the questionnaire;
(c) Provision of informed consent by a parent or legal guardian.

Adolescents with and without a self-reported diagnosis of ADHD were eligible to participate. ADHD diagnostic status was not independently verified through clinical records. No formal exclusion criteria were applied beyond failure to meet the inclusion criteria or incomplete questionnaire responses.

Data were collected using an anonymous, web-based questionnaire that was distributed electronically. Parents were initially contacted by the research team and received detailed information about the study along with consent forms. Adolescents were invited to participate only after written parental consent had been obtained and subsequently provided their own informed assent prior to completing the questionnaire where they were informed they could withdraw at any time. The questionnaire was completed online, allowing participants to respond independently and anonymously. No personally identifiable information (e.g., names or contact details) was collected, and all responses were recorded anonymously. Demographic variables such as gender and age were treated as non-sensitive and were collected solely within the context of anonymized survey research. Parents were instructed not to assist adolescents in answering the questionnaire. Completion of the questionnaire required approximately 10–15 min.

The questionnaire was developed specifically for the purposes of the present study and was not a previously validated instrument. The questionnaire collected information on demographic characteristics, including age, gender, and educational level (Gymnasium, lower secondary education, Grades 7–9, ages 12–15; or Lyceum, upper secondary education, Grades 10–12, ages 16–18), as well as self-reported ADHD diagnosis. Participants were asked about their prior awareness and basic knowledge of ADHD, including familiarity with the disorder and recognition of its core characteristics.

Adolescents’ attitudes and perceptions toward ADHD-related behaviors were assessed using a series of statements describing behaviors commonly associated with ADHD in classroom and social contexts. Responses were recorded on five-point Likert-type scales ranging from strongly disagree to strongly agree.

The questionnaire also examined perceived educational support and intervention, including adolescents’ views on factors that may contribute to improved academic performance and behavior among students with ADHD, such as teacher support, specialized educational or therapeutic services, and individual effort. Adolescents who reported an ADHD diagnosis were additionally asked about their personal experiences with educational or therapeutic support.

The questionnaire comprised sections that differed by participant group. Adolescents without a reported ADHD diagnosis completed items assessing attitudes and perceptions toward classroom behaviors of students with ADHD. Adolescents who reported an ADHD diagnosis completed additional items focusing on their personal experiences, including use of educational or therapeutic support and peer relationships.

Questionnaire sections were completed selectively based on participants’ self-reported ADHD diagnostic status, in accordance with the questionnaire structure (Appendix A). Accordingly, attitude and perception items were completed only by typically developing adolescents, whereas sections regarding personal experiences and support were completed by adolescents with ADHD.

Data were analyzed using SPSS statistics version 28.0 (IBM Corporation, Armonk, NY, USA). Descriptive statistics (means, standard deviations, frequencies, and percentages) were used to summarize participant characteristics and questionnaire responses. Comparisons between adolescents with and without a reported ADHD diagnosis were conducted using chi-square tests. Exploratory Spearman’s ρ rank-order correlation analyses were conducted among selected attitude items completed by typically developing adolescents to examine associations between perceptions of classroom behavior, academic demands, and support needs. Statistical significance was set at *p* < 0.05. No a priori sample size calculation was conducted, as this study was exploratory in nature and aimed to provide descriptive baseline data on adolescents’ knowledge and attitudes toward ADHD. For χ^2^ analyses involving gender, only participants who reported their gender were included. Direct between-group comparisons on attitude items were not conducted, as adolescents with and without ADHD completed different sets of questionnaire items reflecting distinct perspectives.

## 3. Results

### 3.1. Participant Characteristics

A total of 154 adolescents completed the questionnaire. Table 1 shows the sociodemographic characteristics of the sample. Participants were aged 12–18 years, with a mean age of 14.8 years. Regarding educational level, 66/154 (42.9%) were attending Gymnasium (ages 12–15), and 88/154 (57.1%) were attending Lyceum (ages 16–18).

With respect to gender, 59/154 (38.3%) participants identified as male, 90/154 (58.4%) as female, and 5/154 (3.2%) did not report gender.

Based on self-reported diagnostic status, the majority of participants were adolescents of typical development (87%), while 13% reported having a diagnosis of ADHD.

### 3.2. Awareness of ADHD

Overall, 103/154 (66.9%) adolescents reported that they were familiar with ADHD prior to reading the questionnaire description, whereas 51/154 (33.1%) reported no prior knowledge.

Awareness of ADHD did not differ significantly by educational level. Among Gymnasium students, 42/66 (63.6%) reported prior awareness, compared with 61/88 (69.3%) of Lyceum students. This difference was not statistically significant (χ^2^(1) = 0.32, *p* = 0.570).

### 3.3. Perceptions of ADHD-Related Classroom Behaviors Among Typically Developing Adolescents

In addition to identifying classroom behaviors commonly associated with ADHD, typically developing adolescents were asked to select descriptors reflecting their broader perceptions of children with ADHD. Typically developing adolescents (*n* = 134) frequently reported the presence of behaviors associated with ADHD that diverge from typical classroom expectations. The most commonly reported behaviors were becoming distracted in the presence of external noise (83.0%), interrupting the lesson with off-topic questions (70.4%), and getting up from the seat without permission (60.0%).

Other commonly reported behaviors included fatigue or boredom during the lesson (57.8%), talking to classmates during class time (51.9%), and difficulty completing homework assignments (37.4%).

Among typically developing adolescents (*n* = 134), the most common descriptor for a child with ADHD was “has a lot of energy” (126/134, 94.0%). Fewer adolescents selected socially positive classroom descriptors such as “is polite to other children and teachers” (41/134, 30.6%) and “is pleasant in class” (41/134, 30.6%). Reports of classroom self-regulation behaviors were less frequent, including “answers the teacher’s questions when called on” (26/134, 19.4%), “raises his/her hand to speak in class” (16/134, 11.9%), “often does homework” (15/134, 11.2%), and “is quiet during the lesson” (12/134, 9.0%).

### 3.4. Attitudes Toward ADHD-Related Classroom Behaviors

As shown in Table 2, typically developing adolescents expressed mixed attitudes toward classroom behaviors of students with ADHD. A clear majority agreed or strongly agreed that students with ADHD can participate in the classroom (75.5%), indicating generally positive views toward inclusion. At the same time, substantial proportions recognized behavioral challenges, with 58.5% agreeing or strongly agreeing that students with ADHD interrupt lessons more often and 67.4% reporting increased talking during instructional time. Academic difficulties were also frequently reported, as 65.9% agreed or strongly agreed that students with ADHD have lower academic performance and 65.9% reported more frequent failure to complete homework. Neutral responses were common across several items, particularly regarding whether students with ADHD receive sufficient teacher attention (34.8%) and whether they need additional tutoring outside school (28.9%), suggesting uncertainty or ambivalence among respondents. Overall, the findings indicate a coexistence of supportive attitudes toward participation alongside recognition of behavioral and academic challenges associated with ADHD. Adolescents with a self-reported ADHD diagnosis did not complete these items, in accordance with the questionnaire design.

Beyond attitudes toward classroom behaviors, adolescents were also asked about their perceptions of teachers’ responses to students with ADHD. More than half of typically developing adolescents noticed differential treatment by teachers toward a child with ADHD compared with other children (79/134, 59.0%), while 55/134 (41.0%) reported no such difference. Consistent with this, most adolescents supported the view that teachers should provide more attention to a child with ADHD (111/134, 82.8%). A smaller proportion indicated that teacher attention should be the same for all children (17/134, 12.7%), while 6/134 (4.5%) provided other responses (e.g., “it depends” or suggesting additional support without making it conspicuous).

Exploratory Spearman’s ρ rank-order correlations were conducted among five selected attitude items completed by typically developing adolescents (*n* = 134) to examine associations between perceptions of classroom behavior, academic demands, and support needs regarding peers with ADHD. As shown in Table 3, all five items were positively and moderately intercorrelated, with correlation coefficients ranging from ρ = 0.36 to 0.51 (all *p* < 0.001). Greater perceived difficulties in classroom behavioral regulation (e.g., talking more during lessons, difficulty waiting one’s turn to speak) were associated with greater perceived academic difficulties, including difficulty completing homework. Perceptions of greater academic difficulty in peers with ADHD were also associated with a greater perceived need for additional support outside school. Due to the exploratory nature of these analyses, findings should be interpreted cautiously.

### 3.5. Views on Educational Support and Intervention

In addition to attitudes and perceptions of classroom behaviors, adolescents were asked about their broader beliefs regarding whether successful school functioning in ADHD depends primarily on intervention or personal effort. Most participants expressed the view that successful school functioning for students with ADHD depends primarily on teacher attention and individualized intervention by specialists, rather than solely on personal effort. Overall, 122/154 (79.2%) selected “with intervention,” while 32/154 (20.8%) selected “personal effort.” This view differed significantly by gender; analyses included only participants who reported their gender. Among boys, 64.4% selected intervention, compared with 88.9% of girls, a difference that was statistically significant (χ^2^(1) = 11.52, *p* = 0.00069). By educational level, intervention was selected by 74.2% of Gymnasium students and 83.0% of Lyceum students.

Comparisons based on diagnostic status indicated that 110/134 (82.1%) of adolescents without ADHD selected intervention, compared with 12/20 (60.0%) of adolescents with ADHD. This difference was statistically significant (χ^2^(1) = 3.90, *p* = 0.048).

### 3.6. Speech-Language Therapy Perceptions and Uptake

Perceptions of speech-language therapy were examined among typically developing adolescents, while reported uptake and support experiences were assessed among adolescents with ADHD. Among typically developing adolescents (*n* = 134), perceptions of the usefulness of speech-language therapy for peers with ADHD were generally positive. Specifically, 55.6% reported that speech-language therapy could help “to a large extent,” and 46.8% agreed that school performance could improve with appropriate speech-language intervention. In contrast, 27.4% reported not knowing the role of speech-language therapy, and 3.6% believed it would not be helpful.

Among adolescents with ADHD (*n* = 20), the majority reported not receiving speech-language therapy (76.9%), while 23.1% reported current or previous involvement in speech-language therapy.

Regarding support for studying at home, adolescents with ADHD most frequently reported external support (e.g., private tutoring or tutoring center), used by 53.8% of participants. Studying primarily during speech-language therapy sessions was reported by 7.7%, while 38.5% relied mainly on personal effort.

### 3.7. Social Relationships Among Adolescents with ADHD

Most adolescents with ADHD reported positive peer relationships. Specifically, 84% indicated that they had good friends at school and generally positive feelings toward classmates. A smaller number reported having only one close friend and limited interactions with peers.

Regarding social attitudes, most typically developing adolescents reported that impulsive or hyperactive behavior in a peer would not negatively affect them: 43.0% indicated they could become friends with such a peer and 32.6% reported that it would not affect them at all. A smaller proportion (17.0%) reported some social hesitation, while only 5.2% stated that they would not like this behavior.

## 4. Discussion

This study examined Greek adolescents’ knowledge and attitudes toward ADHD, focusing on awareness, perceptions of classroom behaviors, and views on educational support and intervention. Overall, awareness of ADHD was moderate, and typically developing adolescents recognized a range of behaviors commonly discussed in relation to ADHD in classroom settings. Most adolescents expressed supportive views toward intervention and teacher involvement, while adolescents with a self-reported ADHD diagnosis were more likely to emphasize personal effort. Perceptions of speech-language therapy were generally positive, although reported use of such services among adolescents with ADHD was limited.

### 4.1. Awareness and Recognition of ADHD-Related Behaviors

Approximately two thirds of adolescents reported prior awareness of ADHD, indicating a moderate level of familiarity in this sample. Similar levels of awareness have been reported in other adolescent and youth populations, suggesting that ADHD is a relatively visible condition but not necessarily well understood [8,11].

Typically developing adolescents frequently reported behaviors such as distraction by external noise, talking during lessons, and difficulty completing homework. These behaviors overlap with those commonly described in ADHD diagnostic criteria [1], but they are also common features of everyday classroom experience. Importantly, the present findings are descriptive and do not allow conclusions about how adolescents interpret or explain these behaviors. Rather, the results indicate that such behaviors are widely recognized in school settings, without implying that they are perceived as specific to ADHD.

This distinction between recognizing behaviors and understanding their causes is important, as awareness alone does not ensure accurate knowledge and may coexist with uncertainty or misconceptions [8,9]. This distinction was evident in the present study, as only 56% of typically developing adolescents identified ADHD as a neurodevelopmental disorder, while substantial proportions endorsed misconceptions such as poor parenting as a cause (24%) or ADHD being overdiagnosed (59%).

### 4.2. Attitudes Toward Classroom Participation and Behavioral Challenges

Adolescents generally expressed supportive attitudes toward the inclusion of students with ADHD in classroom activities. Most agreed that students with ADHD can participate in the classroom, even while acknowledging behavioral and academic challenges. This pattern suggests that recognition of difficulties does not necessarily translate into exclusionary attitudes, a finding that aligns with previous work highlighting the coexistence of inclusive beliefs and concern about classroom disruption [13,16].

At the same time, neutral responses were common for several items, particularly those related to teacher attention and additional educational support. This may reflect uncertainty rather than negative attitudes, indicating that some adolescents are unsure about what constitutes appropriate support for students with ADHD within the school context. Recent research highlights the central role of the student-teacher relationship in shaping both academic engagement and social adjustment for students with ADHD, suggesting that ambiguity around teacher involvement may reflect limited visibility of these relational processes from the student perspective [21,22].

### 4.3. Views on Intervention and Personal Effort

Most adolescents endorsed the view that successful school functioning for students with ADHD depends on intervention and teacher support rather than personal effort alone. This finding aligns with evidence emphasizing the importance of school-based and multimodal interventions for improving academic and behavioral outcomes in ADHD [4,17].

In contrast, adolescents with a self-reported ADHD diagnosis were more likely to emphasize personal effort. Although this result should be interpreted cautiously, it may reflect increased personal responsibility or pressure experienced by adolescents with ADHD. Previous research has shown that adolescents with ADHD may internalize expectations to manage difficulties independently, which can be associated with self-blame and internalized stigma [10]. While personal effort is important for all students, adequate educational and professional support remains essential.

### 4.4. Perceptions of Speech-Language Therapy and Support Use

Typically developing adolescents generally viewed speech-language therapy as potentially helpful for peers with ADHD, particularly with regard to learning and school performance. However, a substantial proportion reported uncertainty about the role of speech-language therapy, suggesting limited knowledge of available support services. It should be noted that speech-language therapy was examined as one example of specialist educational support within a broader multidisciplinary framework, and the present findings should not be interpreted as suggesting that such intervention is indicated for all adolescents with ADHD.

Among adolescents with ADHD, most reported no current or past involvement in speech-language therapy. Similar gaps between perceived usefulness and actual service use have been reported in other studies of ADHD-related support, often reflecting unmet needs, limited information, or barriers to access [9]. As the present study did not assess reasons for non-use, these findings should be interpreted descriptively.

### 4.5. Social Relationships and Peer Attitudes

Most adolescents with ADHD reported positive peer relationships, and most typically developing adolescents indicated that impulsive or hyperactive behavior in a peer would not negatively affect them socially. Only a small proportion expressed clearly negative reactions. These findings suggest that overt social rejection was not commonly endorsed in this sample, although more subtle social difficulties may not be fully captured by self-report measures [6].

Recent research indicates that peer relationships in ADHD are complex and context-dependent, with friendship quality, peer dynamics, and classroom interactions playing an important role in social adjustment over time [23,24]. Longitudinal evidence further suggests that peer functioning difficulties can both influence and be influenced by ADHD-related behaviors, highlighting the bidirectional nature of these processes [25].

### 4.6. Implications and Limitations

Taken together, the findings suggest that Greek adolescents are broadly aware of ADHD, recognize behaviors commonly associated with it, and are generally supportive of intervention and teacher involvement. At the same time, uncertainty about specific support services and differences in how adolescents with ADHD view responsibility for school functioning highlight areas where clearer information and guidance may be beneficial. By focusing on adolescents’ own reports, the present study adds student-reported data from a Greek secondary school context, an area that appears underrepresented compared with the existing emphasis on educators’ perspectives. Although a study by Pérez-Jorge et al. [26] focused on primary school students, it similarly reported that peers readily recognized ADHD-related behaviors, while attitudes toward classmates with ADHD were not uniformly negative. This convergence suggests that recognition of such behaviors and mixed peer responses may already be present earlier in schooling, although the age groups and study aims differ.

Several limitations should be acknowledged. The cross-sectional design precludes causal interpretations. ADHD status was self-reported and not clinically verified, which may be subject to reporting bias. In addition, comorbid neurodevelopmental or learning conditions (e.g., autism spectrum disorder or specific learning difficulties) were not assessed and SEN categorization was therefore not examined. The sample was obtained through convenience sampling in a single geographic region, which may have introduced selection bias limiting generalizability. Attitudes were assessed using a study-specific, ad hoc questionnaire, and psychometric properties such as reliability and validity were not established. This may have introduced measurement bias. Furthermore, the relatively small size of the ADHD subgroup reflects the pilot nature of the study and limits statistical power for between-group comparisons.

Future research should include larger and more diverse samples, validated measures of ADHD knowledge and stigma, and qualitative approaches to better understand adolescents’ perspectives. School-based educational initiatives that provide clear, age-appropriate information about ADHD and available supports may help address remaining gaps in knowledge and reduce uncertainty. In addition, longitudinal designs incorporating standardized assessment of ADHD symptom severity and comorbid neurodevelopmental or learning conditions would allow for a more comprehensive examination of adolescents’ educational experiences and support needs over time.

## 5. Conclusions

This study examined Greek secondary school students’ knowledge and attitudes toward ADHD, with particular attention to awareness, perceptions of classroom behaviors, and views on educational support. Overall, awareness of ADHD was moderate, and typically developing adolescents recognized several behaviors commonly discussed in relation to ADHD in school contexts. Most adolescents indicated that educational support and teacher involvement play an important role in supporting students with ADHD.

Adolescents with a self-reported ADHD diagnosis were more likely to emphasize personal effort, suggesting differences in how responsibility for school functioning is perceived across groups. While speech-language therapy and other forms of specialist support were generally viewed positively, reported use of such services among adolescents with ADHD was limited.

At the same time, recognition of ADHD-related behaviors did not always correspond to a deeper understanding of the condition or of the supports available within the school setting. These findings suggest that adolescents hold generally supportive views toward peers with ADHD, while some uncertainty remains regarding available supports and their role in the school setting. Schools may therefore represent an appropriate context for providing clear, age-appropriate information about ADHD and educational support options. Further research using larger and more diverse samples is needed to extend these findings.

## Figures and Tables

**Table 1:** Sociodemographic characteristics of the study sample (*n* = 154).

Variable	Statistic	Values
Age (years)	Mean ± SD	14.8 ± 2.06
	Range	12–18
Gender, *n* (%)	Male	59 (38.3)
	Female	90 (58.4)
	Not reported	5 (3.2)
Educational level, *n* (%)	Gymnasium	66 (42.9)
	Lyceum	88 (57.1)
Self-reported ADHD diagnosis, *n* (%)	Yes	20 (13.0)
	No	134 (87.0)

Note: SD refers to standard deviation. Percentages may not total 100 due to rounding.

**Table 2:** Adolescents’ attitudes toward classroom behaviors of students with ADHD (percentages).

Classroom Behavior	Strongly Agree	Somewhat Agree	Neither	Somewhat Disagree	Strongly Disagree
Can participate in classroom activities	45.9	29.6	16.3	2.2	5.9
Difficulty following classroom rules	11.9	30.6	23.9	19.4	14.9
Interrupts lessons more often	24.4	34.1	23.0	14.8	3.7
Talks more during lessons	30.4	37.0	21.5	7.4	3.7
Difficulty completing homework	33.3	32.6	21.5	5.9	6.7
Lower academic performance	35.6	35.6	15.6	5.2	8.1
Difficulty waiting one’s turn to speak	32.6	35.6	19.3	7.4	5.2
Does not receive sufficient teacher attention	15.6	24.4	34.8	13.3	11.9
Needs additional educational support	21.5	32.6	28.9	9.6	7.4

Note: Table responses reflect typically developing adolescents only. Adolescents with a self-reported ADHD diagnosis did not complete these items in the questionnaire. Values are presented as percentages of respondents selecting each response option. Percentages may not total 100 due to rounding.

**Table 3:** Spearman’s ρ rank-order correlations among selected attitude items of typically developing adolescents regarding peers with ADHD (*n* = 134).

Item	1	2	3	4	5
1. Difficulty following classroom rules	—				
2. Talks more during lessons	0.44 ***	—			
3. Difficulty completing homework	0.41 ***	0.51 ***	—		
4. Difficulty waiting turn to speak	0.47 ***	0.42 ***	0.46 ***	—	
5. Needs additional support outside school	0.38 ***	0.36 ***	0.48 ***	0.40 ***	—

Note: All correlations were positive and statistically significant. *** *p* < 0.001.

## Data Availability

The data presented in this study are available on request from the corresponding author due to privacy or ethical restrictions.

## Abbreviations

The following abbreviations are used in this manuscript:

ADHD	Attention-Deficit/Hyperactivity Disorder
APA	American Psychiatric Association

## Appendix A

The 24-item Questionnaire used in this study, translated into the English language, is presented below.

### Adolescents’ Knowledge and Opinions About Attention-Deficit/Hyperactivity Disorder (ADHD) in School

Attention-Deficit/Hyperactivity Disorder (ADHD) is a condition that can affect how people pay attention, control their impulses, and manage their activity levels. These difficulties can sometimes be noticed in the school environment, where students are expected to concentrate, follow rules, and work with others.

ADHD can influence learning, school performance, and relationships with classmates. With the support of trained professionals and appropriate strategies, many of these difficulties can be reduced.

This questionnaire asks about your knowledge and opinions about ADHD and about people who may have difficulties with attention and concentration. There are no right or wrong answers; what matters is your honest opinion.

Your answers will be anonymous, meaning that no one will be able to identify you from your responses, and they will be used only for research purposes. Completing this questionnaire will take about 10–15 min.

This questionnaire is intended for students in Gymnasium and Lyceum. All questions with an asterisk (*) are required.

Section 1 General Information

1. Age * (Free Response)
2. What is your gender? * (Male/Female/Prefer not to say)
3. Level of education? * (Gymnasium/Lyceum)
4. Did you know what ADHD was before reading the introduction of the questionnaire? * (I did know/I did not know)
5. A child with ADHD can function effectively in the classroom if… * (it tries harder/it receives intervention from specialist professionals and more attention from teachers)
6. Do you know someone with ADHD (not yourself)? * (Yes/No)
7. Have you been diagnosed with ADHD? (This question refers to a diagnosis by a healthcare professional.) * (Yes/No)

Section 2 (this was answered by those that answered Yes in Question 7)

8. How much time in total do you spend studying at home per day? * (I don’t study at home at all/Less than 30 min/30–60 min/More than 60 min)
9. Your studying is done with the help of… (you may choose more than one answer) * (My parents/A tutoring center (frontistirio)/A private tutor at home/During Speech and language therapy sessions/I study on my own/Other: ______)
10. Do you attend speech and language therapy? * (Yes/No)
11. Do you think that speech and language therapy can help you, or another child with ADHD, with school performance? * (Yes/No)
12. Do you have good friends at school? * (Yes/No)
13. How do you feel at school in relation to your classmates? * (Free text response)
14. How do you feel at school in relation to your teachers? * (Free text response)
15. Would you like to add any additional comments about the research? (Free text response)

Section 3 (this was answered by those that answered No in Question 7)

16. Please indicate your opinion on the following statements. (The following statements describe possible experiences. Not all children with ADHD experience the same difficulties) *
  Response options for each statement: Strongly agree/Somewhat agree/Neither agree nor disagree/Somewhat disagree/Strongly disagree
  Statements:
  - Children with ADHD can participate in classroom activities.
  - Children with ADHD may have more difficulty following classroom rules compared with other children.
  - Children with ADHD may have difficulty maintaining attention and may interrupt more often during class than other children.
  - Children with ADHD may talk more than other children to the classmate sitting next to them during the lesson.
  - Children with ADHD may have difficulty completing homework as often as other children.
  - Children with ADHD may experience more challenges in their academic performance than other children.
  - Children with ADHD may find it harder to wait for their turn to speak compared with other children.
  - Children with ADHD may not always receive the attention they need from teachers.
  - Children with ADHD may need additional support outside regular school lessons.
17. If a child in your school environment had impulsive behavior or hyperactivity, would that affect you? (choose only one answer). *
  - It would not affect me at all.
  - No, I could become his/her friend.
  - No, but I would not easily become his/her friend.
  - Yes, I would not like this behavior.
  - Other: ______
18. Indicate which of the following describe a child with ADHD (more than one answer allowed). *
  - Has a lot of energy.
  - Is polite to other children and teachers.
  - Is pleasant in class.
  - Is quiet during the lesson.
  - Answers the teacher’s questions when called on.
  - Often does homework.
  - Raises his/her hand to speak in class.
19. Indicate which of the following describe a child with ADHD (more than one answer allowed). *
  - Interrupts the teacher more than other children with questions that are not related to the lesson.
  - Talks more than other children with the classmate sitting next to him/her.
  - Gets bored with the lesson more easily than other children.
  - Gets up from his/her desk more often than other children during the lesson.
  - Forgets homework more often than other children.
  - Has his/her attention distracted by external noise more than other children.
20. In your opinion, how can speech and language therapy help a child with ADHD? (more than one answer allowed). *
  - It will improve his/her performance at school.
  - It will help him/her overcome learning difficulties.
  - I do not know whether speech and language therapy can help.
  - Speech and language therapy will not help with any of the above.
21. Have you noticed different treatment by teachers toward a child with ADHD compared with other children? * (Yes/No)
22. Could teachers give more attention to a child with ADHD? * (Yes, they should give more attention/No, attention should be the same for all children/Other: ______)
23. Does the teacher make more remarks to the child with ADHD because he/she does not pay attention in class? * (Yes/No)
24. Would you like to add anything further about the research? (Free text response).
